# Auditing in addition to compliance monitoring: a way to improve public health

**DOI:** 10.1007/s00038-019-01291-4

**Published:** 2019-08-29

**Authors:** Tine Bizjak, Branko Kontić

**Affiliations:** 1 Department of Environmental Sciences, Jožef Stefan Institute, Jamova cesta 39, 1000 Ljubljana, Slovenia; 2 Jožef Stefan International Postgraduate School, Jamova cesta 39, 1000 Ljubljana, Slovenia

The success of public health policies has recently been questioned in the editorial of the International Journal of Public Health (Gulis 2019), where the author discusses diverse levels of compliance to national health policies and related international agreements. He points out the enduring lack of compliance within countries and concludes with a call for contributions that reveal reasons for this situation. The issue certainly deserves wider attention. In this view, we contribute a deliberation aimed at stimulating national responsible and competent institutions and authorities to consider regular, systematic auditing of public health policies, with the aim of achieving increased policy effectiveness.

The actual effect a public health policy has on a society is determined by the degree of its implementation, which is often unknown or uncertain, due to the scarcity of evidence (Donkor et al. 2018; Kaur 2010). European Commission recently emphasized the importance of responsible application of the EU law (EC 2018). In this context, however, the question of compliance with existing international and national policies remains unanswered, although all EU member states have the same rules, the same access to knowledge, methods and tools (Gulis 2019). Despite differences in political systems, there is a universal condition for ensuring good implementation of health policies: it is the existence of a functional system of active, responsible and competent authorities, who have a clear view about priority areas in the actual public health policy and the power to effectively distribute the available budget. Such a system inevitably includes continuous evaluation of the success of the implementation, focuses on the degree of goals achieved and identifies barriers and drivers encountered in order to make policy improvements.

It is unrealistic to expect intrinsic, successful policy implementation just because it will benefit society. Monitoring the implementation is often limited to collecting general information on the performance and status of activities covered by the policy (Donkor et al. 2018; Usmanova and Mokdad 2013), without thorough evaluation and linkage of the findings to the primary purpose of the monitoring—early alert about needs for policy improvement. Consequently, monitoring can be deficient in terms of identifying barriers and drivers contributing to policy performance. Effective follow-up will continually be needed and should ideally be an integral part of the policy from its planning stages. This follow-up can be performed internally or externally in the form of an audit with appropriate depth and comprehensiveness. Audits generally aim at determining the ways and possibilities of improving a situation in a subject area, checking both the substantial status of the audited subject and the system of actions and measures aimed at achieving its quality. Audits are usually accompanied by contextual questions: “Do we (really) do what we say we do?” and “How good are we in what we are doing?” The answers are used as a non-exhaustive source of improvement measures.

During the past several decades, the inclusion of health into all policies has been repeatedly recognized and advocated (Ståhl et al. 2006; WHO 2017). EU level studies, which review the application and effectiveness of the strategic environmental assessment (SEA) and environmental impact assessment (EIA) directives (Directive 2001/42/EC; Directive 2014/52/EU), also collect information on how health impacts are evaluated in these frameworks. They show that health issues are dealt with unsystematically in the assessments (EC 2016). Regrettably, while recommendations for improving the effectiveness, relevance and coherence of SEA (EC 2016) are included, there are no recommendations for improvement in the area of health impact assessment (HIA). Most recent activities by the WHO Europe again strive to improve the situation in this complex and demanding area (WHO 2019). Additionally, on-going national and international research aims to develop better insight into these issues and their resolution, e.g. within the two EU projects: Integrating Environment and Health Research: a Vision for the EU-HERA (https://www.heraresearcheu.eu/), and Exploring the Neurological Exposome—NEUROSOME (http://www.neurosome.eu/). HERA’s goal is to create a European Health and Environment Research Agenda for 2020–2030, based on the analysis of actual content and gaps in international and national strategies, policies, priorities and research. NEUROSOME focuses on issues related to exposure assessment and neurodevelopmental disorders. Results of these endeavours are expected to help bridge the “know-do” gap and to improve evidence-based health policy development and implementation (van den Driessen et al. 2015; Gulis 2019).

Audits in environmental and industrial safety have already proved effective in protecting the environment and safeguarding economic and occupational health interests. Why should it not be so with regular public health policy audits at the EU and national levels? Besides evaluating the implementation status of the policy, the audits may also effectively check the compliance and adherence on one side and the accountability and honesty of those involved in policy development and implementation on the other. Striving for overall system transparency should be an imperative. In this way, the audits may well prove a success story in contributing to the improved public health status in Europe.
